# *In vivo* Assessment of Supra-Cervical Fetal Membrane by MRI 3D CISS: A Preliminary Study

**DOI:** 10.3389/fphys.2020.00639

**Published:** 2020-06-25

**Authors:** Wenxu Qi, Peinan Zhao, Wei Wang, Zhexian Sun, Xiao Ma, Hui Wang, Wenjie Wu, Zichao Wen, Zulfia Kisrieva-Ware, Pamela K. Woodard, Qing Wang, Robert C. McKinstry, Yong Wang

**Affiliations:** ^1^Department of Obstetrics and Gynecology, School of Medicine, Washington University in St. Louis, St. Louis, MO, United States; ^2^Mallinckrodt Institute of Radiology, School of Medicine, Washington University in St. Louis, St. Louis, MO, United States; ^3^Department of Biomedical Engineering, McKelvey School of Engineering, Washington University in St. Louis, St. Louis, MO, United States; ^4^Department of Physics, Washington University in St. Louis, St. Louis, MO, United States; ^5^Department of Electrical and Systems Engineering, Washington University in St. Louis, St. Louis, MO, United States

**Keywords:** amnion, chorion, fetal membrane, preterm birth, premature rupture of membranes, preterm premature rupture of membranes, magnetic resonance imaging

## Abstract

In approximately 8% of term births and 33% of pre-term births, the fetal membrane (FM) ruptures before delivery. *In vitro* studies of FMs after delivery have suggested the series of events leading to rupture, but no *in vivo* studies have confirmed this model. In this study, we used a three-dimensional constructive interference in steady state (3D-CISS) sequence to examine the FM at the cervical internal os zone during pregnancy; 18 pregnant women with one to three longitudinal MRI scans were included in this study. In 14 women, the FM appeared normal and completely intact. In four women, we noted several FM abnormalities including cervical funneling, chorioamniotic separation, and chorion rupture. Our data support the *in vitro* model that the FM ruptures according to a sequence starting with the stretch of chorion and amnion, then the separation of amnion from chorion, next the rupture of chorion, and finally the rupture of amnion ruptures. These findings hold great promise to help to develop an *in vivo* magnetic resonance imaging marker that improves examination of the FMs.

## Introduction

During pregnancy, the fetus is surrounded by amniotic fluid contained within a fetal membrane (FM). FM is composed of the amnion, which faces the fetus, and the chorion, which contacts the maternal decidua. In a healthy pregnancy, the FM is critical for maintaining a pregnancy until delivery (Parry and Strauss, 1998; Menon and Richardson, 2017). However, in about 8% of pregnancies, the FM ruptures before labor, which is called premature rupture of membranes (PROM). FM rupture before 37 weeks of gestation, termed preterm prelabor rupture of membranes (PPROM), is responsible for approximately one-third of preterm births and is the most common identifiable factor associated with preterm birth (Mathews and MacDorman, 2010; Waters and Mercer, 2011; Martin et al., 2012). Currently, there is no easy way to predict PPROM in early pregnancy, and thus the prevention is very limited.

To solve this problem, we first need to understand the mechanisms of FM rupture. Several investigators have attempted to do so by performing *in vitro* mechanical test on FM after delivery (Artal et al., 1976; Lavery and Miller, 1979; Helmig et al., 1993; Oyen et al., 2004). For example, data from Arikat et al. and Strohol et al. suggest that FM rupture follows this sequence: (1) Amnion and chorion stretch together under load; (2) amnion separates from chorion; (3) chorion ruptures; (4) amnion distends further, non-elastically; and (5) amnion ruptures (Arikat et al., 2006; Strohl et al., 2010). Ultrasound, an imaging modality widely used clinically to monitor pregnancy *in vivo*, can detect some signs associated with PROM and PPROM, such as FM thickness (Frigo et al., 1998; Severi et al., 2008; Başaran et al., 2014; Nunes et al., 2016) and chorioamniotic separation (Devlieger et al., 2003). The FM region that appears to be most prone to rupture is near the internal cervical os (McLaren et al., 1999). However, this para-cervical weak zone is often difficult to visualize by transvaginal ultrasound because of the low contrast between the FM and the maternal decidua (Severi et al., 2008). Strong *in vivo* evidence is still absent in the literature.

Here, we proposed to visualize the FM near the internal cervical os using magnetic resonance (MR) images acquired with a sequence named three-dimensional constructive interference in steady state (3D-CISS). This sequence provides both high spatial resolution and excellent contrast between the cerebrospinal fluid (high signal from water) and tissue structures (lower signal). And thus it is commonly used in clinical procedures to evaluate fine structures, such as cranial nerves surrounded by cerebrospinal fluid (Yoshino et al., 2003; Yousry et al., 2005). In MR images, the difference of signal intensity between amniotic fluid (high signal) and the FM (low/intermediate signal) is similar to the difference of signal intensity between cerebrospinal fluid and nerves, and the FM has similar thickness as nerves. Therefore, the 3D-CISS sequence is able to visualize the FM near the internal cervical os. In our study, we performed 3D-CISS MR imaging on 18 women at one to three time points between 20 and 36 weeks of gestation. And we report the result of four women who had evidence of abnormal FM structure. Our data suggest that the *in vivo* FM rupture sequence matches what proposed from *in vitro* studies (Arikat et al., 2006; Strohl et al., 2010).

## Materials and Methods

### Participants

This study was approved by the Washington University in St. Louis Institutional Review Board (protocols 201612140, 201707152). Participants were recruited by research nurses from the patient population attending the Obstetrics and Gynecology Clinic and the Women’s Health Center in the Barnes-Jewish Hospital Center for Outpatient Health. Participants were included if they were 18 years of age or older and had a healthy singleton pregnancy. Participants were excluded if they had a twin pregnancy or a contraindication to MRI. Before imaging, all patients were screened for MRI safety and provided written informed consent. Age, body mass index, and other clinical information were recorded for all participants. Pregnancy outcomes were collected from the medical records. Term birth was defined as birth between 37 0/7 weeks of gestation and 42 0/7 weeks of gestation (Goldenberg et al., 2008). Preterm birth was defined as birth between 20 0/7 weeks of gestation and 36 6/7 weeks of gestation (Goldenberg et al., 2008). PROM was defined as rupture of membranes before labor. PPROM was defined as rupture of membranes followed by labor before 37 weeks of gestation (Simhan and Canavan, 2005; Goldenberg et al., 2008).

### MRI Acquisition

Every patient underwent MRI examination one, two, or three times between 20 and 36 weeks of gestation. A Siemens Magnetom Vida 3T whole body MRI scanner and a 30-channel phased-array torso coil (Erlangen, Germany) were used to acquire a series of sagittal view T2 weighted images (T2WI), with a half-Fourier acquisition single-shot turbo spin echo sequence and the following parameters: repetition time, 1800 ms; echo time, 94 ms; matrix, 320 × 650; flip angle, 140°; layer thickness, 4.0 mm; slice spacing, 0.8 mm; number of layers, 25. For the 3D-CISS sequence, parameters were as follows: repetition time, 7.71 ms; echo time, 3.70 ms; flip angle, 50°; acquisition number, 1; acquisition matrix, 640 × 640; field of view, 300 mm × 300 mm; bandwidth, 460 Hz per pixel; slice thickness, 1 mm; and in-plane resolution, 0.33 mm × 0.33 mm. The total acquisition time for both T2WI and 3D-CISS was 7 min.

### Image Analysis

Magnetic resonance images were independently analyzed by two radiologists (WQ and WW, with 10-year and 1-year of experience, respectively, in analyzing abdominal MR images) who were blinded to pregnancy outcomes. A consensus was reached in cases of discordance. The following imaging characteristics were evaluated: cervical funneling, chorioamniotic separation, and chorion or amnion rupture.

## Results

Between April 2019 and February 2020, 18 pregnant women were recruited for this study. Their mean age was 33.5 ± 12.1 years, and their mean body mass index at first prenatal visit was 23.8 ± 5.3 kg/m^2^. Demographic and clinical details of the 18 women included in this study are presented in Table 1. A total of 43 MRI scans were performed on these 18 patients.

**TABLE 1:** Demographic and clinical characteristics of pregnant women.

	**Total (*n* = 18)**	**ROM at labor (*n* = 17)**	**PPROM (*n* = 1)**
Age, years, median (range)	26.5 (19–35)	26 (19–35)	25
Body mass index, kg/m^2^, average (range)	27.68 (18.5–39.0)	27.66 (18.5–39.0)	28.0
Race/ethnicity, *n* (%)			
African American	16 (88.9)	15 (88.2)	1 (100)
Caucasian	2 (11.1)	2 (11.8)	0
Asian	0	0	0
Other	0	0	0
Multiparous, *n* (%)	16 (88.9)	15 (88.2)	1 (100)
Nulliparity	2 (11.1)	2 (11.8)	0

**FIGURE 1:**
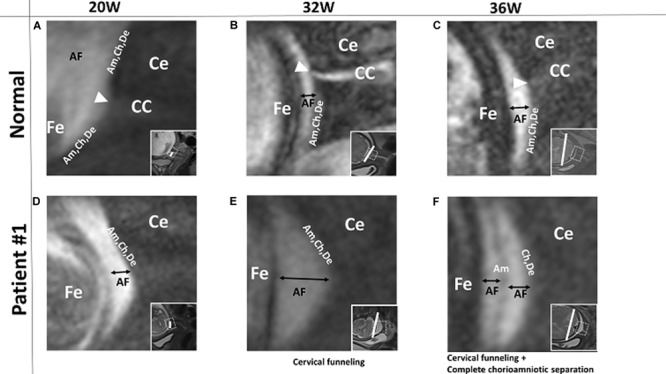
A pregnant woman with normal FM and Patient #1. **(A–C)** 3D-CISS images from a pregnant woman with normal, intact FM at the indicated time points. The white triangle indicates FM suspended in the cervical canal region. Images from patient #1, showing normal FM at 20 weeks **(D)**, cervical funneling at 32 weeks **(E)**, and cervical funneling and complete chorioamniotic separation at 36 weeks **(F)**. Insets show T2WI images of the same regions. The white lines indicate the diameter of the cervix anatomical internal os. AF, amniotic fluid; Am, amnion; CC, cervical canal; Ce, cervix; Ch, chorion; De, decidua; Fe, fetus.

Fourteen patients had normal-appearing FM in which the amnion, chorion, and decidua were intact and indistinguishable from one another at all imaging time points. For example, in the patient images shown in Figures 1A–C, the FM was completely intact at 20, 32, and 36 weeks’ gestation, though we noted some suspended FM material in the cervical canal at all three time points. None of the 14 patients with normal, intact FM had PPROM or PROM, and all 14 delivered at term.

Four patients had both cervical funneling, in which the FM protruded into the cervix, and chorioamniotic separation, in which amniotic fluid was visible between the amnion and chorion, detectable in at least one of their MRI scans.

In patient #1, the FM appeared normal at 20 weeks (Figure 1D). However, at 32 weeks, this patient had cervical funneling with amniotic fluid and FM protruding into the cervix (Figure 1E). At 36 weeks, amniotic fluid was visible between amnion and chorion, indicating chorioamniotic separation (Figure 1F). This patient did not have PPROM or PROM and delivered at term.

**FIGURE 2:**
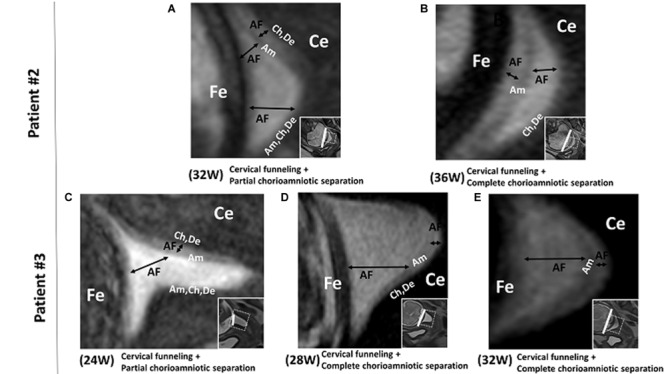
Patients # 2 and # 3. 3D-CISS images from patient #2, showing cervical funneling and partial chorioamniotic separation at 32 weeks **(A)** and cervical funneling and complete chorioamniotic separation at 36 weeks **(B)**. Images from patient #3, showing cervical funneling and partial chorioamniotic separation at 24 weeks **(C)** and cervical funneling and complete chorioamniotic separation at 28 weeks **(D)** and 32 weeks **(E)**. Insets show T2WI images of the same regions. The white lines indicate the diameter of the cervix anatomical internal os. AF, amniotic fluid; Am, amnion; Ce, cervix; Ch, chorion; De, decidua; Fe, fetus.

In patient #2, the FM showed cervical funneling and partial chorioamniotic separation at 32 weeks and complete chorioamniotic separation at 36 weeks (Figures 2A,B). This patient did not have PPROM or PROM and delivered at term.

In patient #3, the FM showed cervical funneling and partial chorioamniotic separation at 24 weeks and complete chorioamniotic separation at 32 and 36 weeks (Figures 2C–E). This patient did not have PPROM or PROM and delivered at term.

**FIGURE 3:**
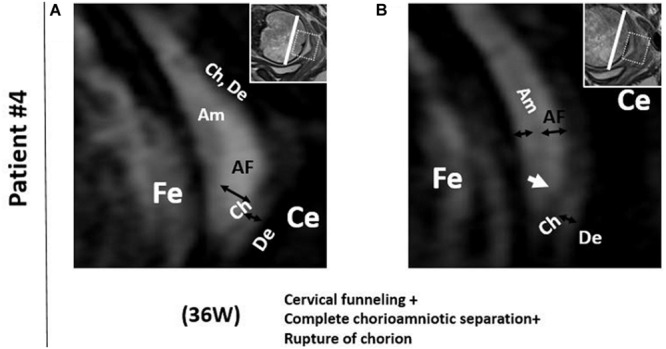
Patient 4. 3D-CISS images from patient #4 showing cervical funneling, chorioamniotic separation, and chorionic rupture at 36 weeks. The white arrow indicates the point of chorionic rupture. Insets show T2WI images of the same regions. The white lines indicate the diameter of the cervix anatomical internal os. AF, amniotic fluid; Am, amnion; Ce, cervix; Ch, chorion; De, decidua; Fe, fetus.

In patient #4, the FM showed deeper cervical funneling, chorioamniotic separation, and chorionic rupture at 36 weeks (Figure 3). This patient developed PPROM 6 h after the MRI scan and delivered preterm (36 2/7 weeks).

## Discussion

**FIGURE 4:**
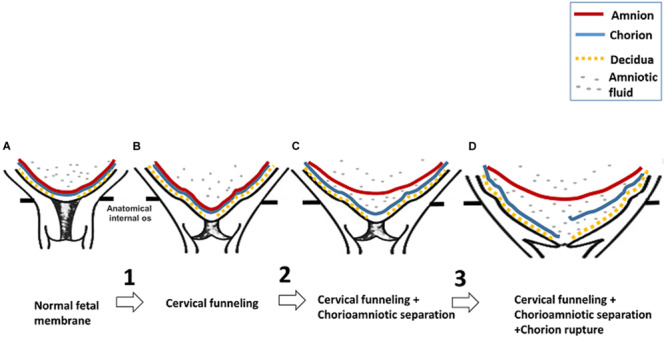
Schematic of the first three steps of premature FM rupture detected by 3D-CISS. **(A)** After 16 weeks’ gestation, the amnion (red) and chorion (blue) usually fuse, and the chorion is fused to the decidua (yellow) at the maternal–fetal interface. No amniotic fluid (gray dots) can be seen between the amnion and chorion or between the chorion and decidua. **(B)** In step 1, the FM stretches as it protrudes into the cervix when the internal cervical os dilates, causing cervical funneling. **(C)** In step 2, the amnion separates from the chorion, and amniotic fluid is detectable between the amnion and chorion. **(D)** In step 3, the FM undergoes additional stretch upon further internal cervical os dilation. This can result in chorion rupture.

In our study, the longitudinal 3D-CISS MRI data provide the first *in vivo* evidence to support the first three steps of the model proposed by Arikat et al. regarding the sequence of events leading to FM rupture and PROM or PPROM. In the first step of their model, the FM stretches and protrudes into the cervix when the cervical internal os dilates to cause cervical funneling. This is evident in patient #1 at 32 weeks. In step 2, the amnion partially or completely separates from the chorion, as is evident in patient #1 at 36 weeks, patient #2 at 32 and 36 weeks, patient #3 at 24, 28, and 32 weeks, and patient #4 at 36 weeks. In step 3, further cervical internal os dilation leads to additional FM stretch and chorion rupture as seen in patient #4 at 36 weeks. In step 4, the amnion distends further. Finally, in step 5, the amnion ruptures, leading to PPROM or PROM. We present a schematic of the first three steps of this model in Figure 4.

Consistent with the *in vitro* studies, our *in vivo* study indicates that the stretch of FM is the first step in FM rupture. During pregnancy, outward pressure on FM from the amniotic fluid is balanced by inward pressure from the uterine wall. However, when the cervical internal os opens (cervical funneling), inward pressure on the FM overlying the cervix will decrease, and the FM will protrude into the cervical canal, causing the stretch of FM. Our longitudinal data suggest that the FM stretch in the para-cervical weak zone can lead to chorioamniotic separation. Data from *in vitro* studies suggest that the mechanical force applied to FM reduces the adhesiveness between amnion and chorion, leading to chorioamniotic separation (Strohl et al., 2010). This result is also supported by *in vitro* second harmonic generation microscopy studies of FM, revealing that the repeated mechanical loading affects the integrity of the amnion–chorion interface and can increase the risk of FM rupture (Mauri et al., 2013).

Before 14 weeks’ gestation, the chorion and amnion have not yet fused together, and the chorioamniotic separation is always normal. After 16 weeks, however, any chorioamniotic separation is identified as uncommon and anomalous (Kim et al., 2007; Bibbo et al., 2016). Such separation is dangerous, as the ultrasound-detected chorionic separation after 16 weeks is associated with adverse perinatal outcomes such as fetal extremity deformities, fetal death (Graf et al., 1997; Levine et al., 1998), and preterm delivery (Levine et al., 1998; Sydorak et al., 2002; Devlieger et al., 2003; Wilson et al., 2003). The 3D-CISS images can detect chorioamniotic separation, since the amniotic fluid lies between the chorion and amnion.

We observed that the chorioamniotic separation which occurs before FM rupture is consistent with three sets of previous data. First, in clinical observations, FM components are frequently separated at delivery after spontaneous rupture of the membranes before delivery (Strohl et al., 2010). Second, a video-recorded sequence of *in vitro* FM rupture revealed that the chorion and amnion separated before rupture (Arikat et al., 2006). Third, in *in vitro* mechanical tests, two peaks were noted in the force vs. displacement curve, suggesting that FM rupture occurs via separate rupture of the amnion and chorion (Artal et al., 1976; Lavery and Miller, 1977; Oxlund et al., 1990; Schober et al., 1994; El Khwad et al., 2005).

In patient 4, the chorion ruptured before the amnion, which is supported by *in vitro* studies (Artal et al., 1976; Lavery and Miller, 1979; Helmig et al., 1993; Oyen et al., 2004; Arikat et al., 2006). But some studies suggest that the amnion ruptures first (Artal et al., 1976; Lavery and Miller, 1979; Helmig et al., 1993; Oyen et al., 2004; Arikat et al., 2006). Our data are consistent with *in vitro* mechanical testing revealing that the amnion was consistently stronger, stiffer, and more ductile than the chorion (Arikat et al., 2006). The amnion may be stronger because it is composed of a dense layer of collagen fibrils, where the FM strength mainly comes from (Strauss, 2013).

The major strength of this work is the first ever use of 3D-CISS MRI to obtain *in vivo* images of the FM at much higher contrast and better resolution than other types of MRI or ultrasound. Clinical ultrasound is a series of 2D images acquired at several limited angles, which cannot provide a 3D description of the FM overlying the cervix. In comparison, 3D-CISS MRI is not operator dependent and can provide a high resolution, high contrast 3D spatial coverage of FM with multi-planar viewing angle capability. Therefore, 3D-CISS MRI provides a novel way to study the FM overlying the cervix. Additionally, by longitudinally imaging patients, we could define the sequences of events leading to FM rupture.

In this study, we used a 3.0 T MRI to image the FM of pregnant women. MRI has been used to evaluate obstetrical, placental, and fetal abnormalities in pregnant patients for more than 30 years, and its application during pregnancy is generally considered safe for the fetus (Patenaude et al., 2014; Radiology TACo, 2015; Ray et al., 2016). Compared with the current commonly used fetal MRI sequence, the 3D-CISS sequence was applied without exceeding either of the specific absorption rate and acoustic noise. Additionally, 3D-CISS is a high-speed sequence (4 min) and therefore reduced the patients’ exposure to the magnetic.

Our study has three main limitations. First, we had a small sample size and our data are qualitative in nature. Second, we did not measure other FM characteristics such as thickness and signal intensity. Lastly, our medical records did not separate PPROM from PTL in the history of preterm delivery.

## Conclusion

In summary, our data support the *in vitro* model that the FM ruptures according to a sequence starting with stretch of the chorion and amnion together, then separation of the amnion from the chorion, next the rupture of the chorion, and finally the rupture of the amnion ruptures. An important next step is to conduct a larger longitudinal study to confirm these findings. If we can define an MRI marker that predicts FM rupture, we may be able to intervene to prevent PPROM.

## Data Availability Statement

All datasets presented in this study are included in the article/supplementary files.

## Ethics Statement

The studies involving human participants were reviewed and approved by the Washington University in St. Louis Institutional Review Board (protocols 201612140 and 201707152). The patients/participants provided their written informed consent to participate in this study.

## Author Contributions

WQ and PZ designed the experiment. WQ and WiW evaluated magnetic resonance images. ZS, XM, HW, WnW, ZW, ZK-W, PW, and QW collected the data and aided in preparation of the manuscript. RM co-supervised the research. YW obtained funding for the project, supervised the work, and participated in preparation of the manuscript.

## Conflict of Interest

The authors declare that the research was conducted in the absence of any commercial or financial relationships that could be construed as a potential conflict of interest.
